# Angiotensin Receptor Blockers Upregulate Angiotensin Type 4 Receptor in Brains of Cognitively Intact Individuals

**DOI:** 10.1093/geroni/igab046.2411

**Published:** 2021-12-17

**Authors:** Caglar Cosarderelioglu, Claudene J George, Qian-Li Xue, Esther Oh, Luigi Ferrucci, David Bennett, Jeremy Walston, Peter M Abadir

**Affiliations:** 1 Ankara University School of Medicine, Cankaya, Ankara, Turkey; 2 Albert Einstein College of Medicine/Montefiore Medical Center, New York City, New York, United States; 3 Johns Hopkins University School of Medicine, Baltimore, Maryland, United States; 4 National Institute on Aging, Baltimore, Maryland, United States; 5 Rush University, Chicago, Illinois, United States

## Abstract

The primary dementia-protective benefits of Angiotensin receptor type 1 (AT1R) blockers (ARBs) are believed to arise from systemic effects on blood pressure. However, there is a brain-specific renin-angiotensin system (b-RAS) that acts mainly through three receptor subtypes: AT1R, AT2R, and AT4R. AT1R promotes inflammation and oxidative stress (OS). AT2R increases nitric oxide. AT4R is essential for dopamine release and mediates memory consolidation. Here, we aimed to investigate the effects of ARBs on b-RAS, OS, inflammation, PHF-tau, and beta-amyloid load. Postmortem frontal-cortex brains of age- and sex-matched cognitively intact (CI) individuals using (n=30) and not using ARBs (n=30) and Alzheimer's disease (AD) patients using (n=30) and not using ARBs (n=30) were studied. Protein levels of receptors were measured by Western blot. Protein carbonyl (PC) and cytokine levels were measured by ELISA. Tangle and amyloid-β scores were used as outcomes. In CI individuals, our data shows that ARB treatment was associated with higher protein levels of AT4R (median(range) 0.69(1.92) vs 0.17(1.18) CI+ARBs vs CI, p=0.02), lower level of OS marker PC (10.60(8.32) vs 11.26(7.44), CI+ARBs vs CI, p=0.03) and lower hippocampal and overall amyloid scores (0(5.45) vs 1.15(4.21) p=0.03, 0.79(12.75) vs 3.41(13.36) p=0.04, CI+ARBs vs CI, respectively). In AD group, ARB treatment was associated with lower AT1R protein levels (0.47(1.15) vs 0.59(1.99), AD+ARBs vs AD, p=0.02). No significant changes were observed in OS, inflammation, or PHF-tau and amyloid load in AD brains treated with ARBs. Our results highlight the impact of ARBs on the brains of cognitively intact and AD older individuals.

